# Preoperative Factors Predicting the Preservation of the Posterior Cruciate Ligament in Total Knee Arthroplasty

**DOI:** 10.1111/os.13439

**Published:** 2022-08-17

**Authors:** Yi Wang, Liyi Zhang, Jianhao Lin, Dan Xing, Qiang Liu, Diange Zhou

**Affiliations:** ^1^ Department of Orthopaedic Surgery Beijing Huairou Hospital of Beijing Traditional Chinese Medicine Beijing China; ^2^ Arthritis Clinic and Research Center Peking University People's Hospital Beijing China; ^3^ Arthritis Institute Peking University Beijing China; ^4^ Beijing Jishuitan Hospital Beijing China

**Keywords:** Knee geometrics, Posterior cruciate ligament, Predictor, Total knee arthroplasty

## Abstract

**Objective:**

Predicting the successful preservation of posterior cruciate ligament (PCL) in total knee arthroplasty (TKA) is an important step for preoperative planning to secure the satisfactory outcomes. We aimed to examine the preoperative factors predicting the successful preservation of the PCL in cruciate‐retaining TKA and the outcome of sacrificing the PCL.

**Methods:**

In this retrospective study, we analyzed TKAs consecutively performed by a single surgeon between January 2019 and August 2021 who had been preoperatively planned to undergo implantation of cruciate‐retaining (CR) prostheses. The outcome of the current study was whether the PCL was retained or sacrificed. Anterior‐stabilized (AS) tibial bearings when the PCL was sacrificed as needed were used intraoperatively. Age, sex, body mass index (BMI), and preoperative diagnosis from the patients' medical records were obtained. The medial‐lateral width of epicondyle (MLW), the medial posterior condyle height (MPCH), the lateral posterior condyle height (LPCH), the ratio of MLW and MPCH, the ratio of MLW and LPCH, the Insall–Salvati index, and the severity of the varus or valgus deformity were measured using preoperative radiographs. Univariate and multivariate regression were fitted to assess the association of these factors with the successful retention of PCL. To examine the influence of sacrifice of the PCL on the surgical procedure, the size of the tibial and femoral components, the thickness of the polyethylene insert, and the rate of patella replacement between the CR group and AS group were also compared using *t* tests or chi‐square tests.

**Results:**

Among 307 TKAs included, PCL was sacrificed with concurrent use of AS prostheses in 89 (29.0%) procedures. Knees with rheumatoid arthritis (*P* < 0.01), lower Insall–Salvati index (*P* < 0.01), and more severe varus deformity (*P* = 0.011) were at a higher risk of sacrificing the PCL intraoperatively. There was no significant difference in age, sex, BMI, MLW, MPCH, LPCH, ratio of MLW and MPCH, ratio of MLW and LPCH, size of the tibial and femoral components, or replacement of the patella between the CR and AS groups. Converting from CR to AS was associated with a higher risk of using a thicker polyethylene insert (*P* < 0.01).

**Conclusion:**

Rheumatoid arthritis, lower Insall–Salvati index, and more severe varus deformity were associated with an increased risk of sacrificing the PCL in TKAs planned to undergo implantation CR prostheses. Converting to AS tibial bearing may result in a thicker polyethylene insert. These factors should be carefully considered for the appropriate selection of prosthesis type preoperatively.

## Graphical abstract

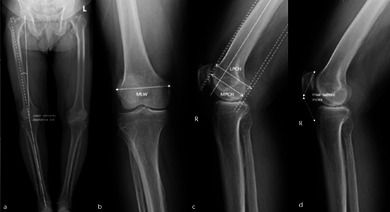

## Introduction

Posterior cruciate ligament (PCL) retaining (CR) and PCL substituting (PS) prostheses are two classical options for total knee arthroplasty (TKA).^1^ The two prostheses have similar results in terms of clinical, functional, radiological outcomes, and complications.^2^ Short‐term and long‐term prosthesis survivorship for both CR and PS TKA is satisfactory.^3^ However, a successful CR TKA requires different surgical techniques from the PS TKA because sacrificing the PCL opens the flexion gap 2 mm more than the extension gap,^4^ For example, surgeons performing CR TKA should begin by removing the less distal femur to keep the extension space equally small. Furthermore, many knee systems currently under use do not allow a conversion from CR to PS.^5^ Therefore, from a technical perspective, as a part of the preoperative plan, it is preferable to make the decision to use CR or PS prostheses preoperatively.

Some surgeons prefer to choose prostheses according to their own experience and preference. Other surgeons may refer to intraoperative gap measurements to decide whether to preserve the PCL.^6^ Nonetheless, surgeons may inevitably face the problem of sacrificing the PCL during the CR TKA. It was reported that rate of intraoperative conversion from CR to PS in TKAs ranged from 9.9% to 17.0%.^7^, ^8^ Even for well‐designed implant systems that allow an intraoperative switch from CR to PS, sacrificing PCL after bone cutting may compromise surgical efficiency and result in undesirable conditions such as a thicker polyethylene insert or posterior flexion instability.^9^, ^10^ Additional efforts must be made to avoid or compensate for these problems. Therefore, it is recommended that surgeons envision the selection between CR and PS in the preoperative plan based on history, physical examination, and imaging and laboratory tests, taking into account both the advantages and disadvantages of implant designs.^11^

Risk factors predicting the outcome of TKA have been widely studied,^12^ which helps surgeons make a sound preoperative plan and modify techniques. Specifically, for CR TKA, the successful preservation of PCL is of utmost importance. However, there is a paucity regarding the preoperative predictors that can help surgeons identify potentially challenging cases of CR TKA and prevent a prolonged duration of surgery and unsatisfactory results. To fill this knowledge gap and address these challenges, we conducted a retrospective study to determine: (i) the rate and risk factors for sacrificing PCL; and (ii) the outcomes of sacrificing the PCL in preplanned CR TKAs.

## Materials and Methods

This retrospective study was approved by the ethics committee of the Peking University People's Hospital (2021PHB431‐001).

### Participants

We retrospectively analyzed 307 knees from 266 patients who underwent primary TKA between January 2019 and August 2021 at Peking University People's Hospital. The inclusion criteria were primary TKAs by a single surgeon who utilized the same prosthesis model allowing the sacrificing of PCL intraoperatively. We excluded those with missing values in the variables of interest, or preoperative or postoperative radiographs. All knees were preoperatively planned to use a CR knee prosthesis and were implanted into the Biomet Vanguard Complete Knee System by one surgeon (DZ). Among them, 254 (82.7%) were women. The average age at the time of TKA was 67.0 ± 7.6 years (range, 33–90 years). The diagnosis was osteoarthritis for 297 knees and rheumatoid arthritis for 10 knees.

### Surgical Technique

All TKAs were performed under tourniquet control using a subvastus approach through a midline skin incision. Bone cuts were made using a measured resection technique. The distal femoral cut was made at 6° valgus angulation using an intramedullary guide. Approximately 7 mm of bone was removed from the distal femur. An increased amount of bone resection was applied when there was a high flexion contracture (>30°). Rotation of the femoral component was determined with reference to the transepicondylar axis. The size of the femoral implant was determined using the anterior‐referencing guide. The proximal tibial cut was made using an extramedullary guide perpendicular to the long axis of the tibia. The tibial posterior slope was usually set to 5°. Medial or lateral soft tissue contracture was manually evaluated carefully and released as needed. Flexion‐extension balance, bilateral stability and range of motion (ROM) were tested using trial components. When flexion tightness was indicated by lift‐off or paradoxical rolling forward, the PCL was further carefully released or the posterior slope of the tibia was increased. If flexion‐extension or mediolateral gaps remained mismatched after these efforts, the PCL was sacrificed, and an anterior‐stabilized (AS) tibial bearing was used. PCL was recessed in 89 (29.0%) knees. The selective patella resurfacing was performed. Components were cemented for all knees.

### Demographics of Participants

We retrospectively obtained age, sex, body mass index (BMI), preoperative diagnosis, size of the tibial and femoral components, and thickness of the polyethylene insert from patients' medical records.

Age was classified into 5 categories (<60, 60–65, 65–70, 70–75, and >75). BMI was classified into≤18.5, 18.5–<24.0, 24.0–<30.0, and ≥30.0 kg/m^2^. The size of the femoral components used in our study was 55, 57.5, 60, 62.5, 65, 67.5, 70, and 72.5 mm. The size of the tibial components used in our study was 63, 67, 71, 75, 79, and 83 mm. The medial‐lateral length of the femoral components for size 55, 57.5, 60, 62.5, 65, 67.5, 70, and 72.5 mm were 59, 61, 64, 66, 68, 71, 73, and 75 mm, respectively. The thickness of the polyethylene insert used in our study were 10, 12, and 14 mm. The thickness of the polyethylene insert used in our study were divided into two groups (10 and >10 mm).

The outcome in the current study was whether the PCL was retained or sacrificed. Patients were thus divided into CR and AS groups.

## Measurement Methods

**Fig. 1:**
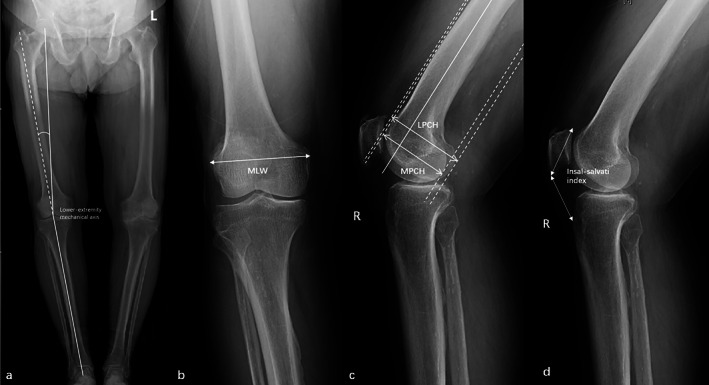
Measurement of the radiograph factors. (A) The mechanical axis was defined as the center of the plateau to the center of the plafond, and the anatomic axis was defined as the center of the tibial diaphysis. (B) MLW: medial‐lateral width of epicondyle. The MLW was defined as the distance between the lateral epicondyle and the medial epicondyles. (C) MPCH: medial posterior condyle height, LPCH: lateral posterior condyle height. The longitudinal posterior condylar line was determined on the cut with the largest anterior–posterior dimension at the lateral and medial femoral condyle. The distance between the anterior femoral cortex line and the posterior condylar line of the lateral and medial femoral condyle was defined as LPCH or MPCH. (D) Insall–Salvati index: The Insall–Salvati index was defined as the Patellar length compared to the patellar tendon length

We defined the medial‐lateral width of epicondyle (MLW) as the length of femoral epicondylar axis on the antero‐posterior standing view of knee. We measured the medial posterior condyle height (MPCH), the lateral posterior condyle height (LPCH), and the Insall–Salvati index using lateral view of knees, and the mechanical axis using full limb radiograph (Fig. 1). These measurements were performed by an orthopedic surgeon (YW) masked from the patients' information using a picture archiving and communication system. The normality of all radiographs was checked.

The lower extremity mechanical axis was classified into severe varus deformity (>15° varus), mild varus deformity (varus between 5° and 15°), neutral position (within 5° varus or valgus), mild valgus deformity (valgus between 5° and 15°), and severe valgus deformity (>15° valgus). The Insall–Salvati index was classified into four quartiles. We calculated the difference between the MLW and the medial‐lateral lengths of the femoral components to reflect the femoral coverage.

### Statistical Analysis

We compared continuous variables, such as MLW, LPCH, the ratio of MLW and MPCH, the ratio of MLW and LPCH, and the difference between the MLW and the medial‐lateral width of the femoral components between the CR and AS groups using Student's *t* test. We fitted the chi‐squared test to compare categorical variables, such as age, sex, preoperative diagnosis, lower‐extremity mechanical axis, the Insall–Salvati index, the size of the tibial and femoral components, the thickness of the polyethylene insert, and the replacement of patella, between the CR and AS groups. We performed univariate analysis to examine the association of risk factors with the successful retention of PCL. We then performed a Poisson regression with a robust variance estimate to further examine the association between preoperative clinical and radiographic factors and the successful preservation of the PCL.^13^ We also performed tests for linear trends by entering the median value of each category of variables of interest as a continuous variable in the models.^14^ A two‐sided *p* value of 0.05 was considered statistically significant. All data were analyzed by IBM® SPSS® Statistics (Version 22.0, IBM, Armonk, NY, USA) software.

## Results

### Characteristics of Participants

Overall, 307 knees were included in this study. Among them, 218 knees were classified into the CR group while 89 knees were classified into the AS group. The mean MLW was 8.37 ± 0.69 cm in the CR group and 8.40 ± 0.74 cm in the AS group. As shown in Table 1, the mean MPCH was 6.33 ± 0.58 cm in the CR group and 6.28 ± 0.63 cm in the AS group. The mean LPCH was 6.30 ± 0.53 cm in the CR group and 6.25 ± 0.58 cm in the AS group. We did not observe any statistically significant difference in the MLW, MPCH or LPCH between the CR and AS group. The mean values of the above parameters according to age and sex are shown in Table 2. There was no significant difference between age groups. Men tended to have larger MLW, MPCH, and LPCH (*P* < 0.001).

**TABLE 1:** Comparison of patients' demographic and clinical factors and radiographic measurements between retaining and recession of posterior cruciate ligament (PCL)

	CR group	AS group	Univariate analysis		Multivariate analysis	
			T/F	*P* value	OR	*p* value
Age*	67.4 ± 7.4	65.8 ± 8.7	5.652	0.227		
<60	26 (%)	14 (%)			–	–
60–65	47 (%)	19 (%)			0.838	0.542
65–70	71 (%)	24 (%)			0.707	0.213
70–75	35 (%)	22 (%)			1.094	0.742
>75	39 (%)	10 (%)			0.548	0.098
Gender*			0.045	0.833		
Male	37 (%)	16 (%)			–	–
Female	181 (%)	73 (%)			0.979	0.927
BMI*	26.87 ± 3.56	26.49 ± 3.76	0.938	0.626		
<=24.0	44 (%)	22 (%)			–	–
24.0–30.0	136 (%)	54 (%)			0.770	0.217
>30.0	38 (%)	13 (%)			0.741	0.322
Preoperative diagnosis*				0.008		
Osteoarthritis	215 (%)	82 (%)			–	–
Rheumatoid arthritis	3 (%)	7 (%)			2.563	<0.001^†^
MLW	8.37 ± 0.69	8.4 ± 0.74	−0.365	0.715		
MPCH	6.33 ± 0.58	6.28 ± 0.63	0.586	0.558		
LPCH	6.3 ± 0.53	6.25 ± 0.58	0.666	0.506		
The ratio of MLW and MPCH	0.76 ± 0.06	0.75 ± 0.06	1.117	0.265		
The ratio of MLW and LPCH	0.75 ± 0.05	0.75 ± 0.05	1.385	0.167		
Insall‐salvati index^‡^			16.488	0.001		
0–1/4	41 (%)	36 (%)			–	–
1/4–2/4	56 (%)	20 (%)			0.636	0.060
2/4–3/4	60 (%)	15 (%)			0.458	0.002^†^
3/4–1	61 (%)	18 (%)			0.526	0.010^†^
*p* for trend					0.100	0.006^†^
Lower‐extremity mechanical axis^‡^				0.027		
>15° varus	25 (%)	20 (%)			2.058	0.012^†^
Varus between 5° to 15°	137 (%)	43 (%)			1.028	0.917
Neutral position	42 (%)	15 (%)			–	–
Valgus between 5° to 15°	10 (%)	7 (%)			1.717	0.122
Valgus >15	4 (%)	4 (%)			1.431	0.385
*p* for trend					0.993	0.606

* Adjusted for age, gender and BMI

^†^ *p* < 0.05

^‡^ Adjusted for age, sex, BMI, and preoperative diagnosis

–: as reference, T: *t* value for *t* test, *F*: *F* value for chi‐squared test, OR: odds ratio, MLW: medial‐lateral width of epicondyle, MPCH: medial posterior condyle height, LPCH: lateral posterior condyle height

**TABLE 2:** Radiological measurements of knee geometrics according to age and sex

	MLW	MPCH	LPCH	The ratio of MLW and MPCH	The ratio of MLW and LPCH	Insal‐salvati index	Lower‐extremity mechanical axis
Age							
<60							
Male	9.59 ± 0.93	6.97 ± 0.22	7.15 ± 0.37	0.74 ± 0.08	0.75 ± 0.06	0.91 ± 0.13	−5.42 ± 6.89
Female	8.26 ± 0.53	6.24 ± 0.47	6.2 ± 0.44	0.76 ± 0.05	0.75 ± 0.05	1.04 ± 0.17	−6.53 ± 8.68
60–65							
Male	9.29 ± 0.49	6.94 ± 0.44	6.85 ± 0.39	0.75 ± 0.05	0.74 ± 0.05	1 ± 0.25	−9.63 ± 4.72
Female	8.13 ± 0.53	6.2 ± 0.55	6.18 ± 0.45	0.76 ± 0.05	0.76 ± 0.05	1.05 ± 0.15	−9.59 ± 5.18
65–70							
Male	9.25 ± 0.58	6.81 ± 0.56	6.76 ± 0.48	0.74 ± 0.05	0.73 ± 0.05	1.03 ± 0.16	−8.22 ± 8.73
Female	8.26 ± 0.57	6.19 ± 0.50	6.17 ± 0.49	0.75 ± 0.06	0.75 ± 0.05	1.05 ± 0.15	−6.71 ± 10.13
70–75							
Male	9.31 ± 0.55	7.12 ± 0.59	7.16 ± 0.40	076 ± 0.04	0.77 ± 0.03	0.95 ± 0.14	−8.51 ± 3.08
Female	8.03 ± 0.58	6.1 ± 0.48	6.02 ± 0.45	0.76 ± 0.06	0.75 ± 0.05	1.04 ± 0.12	−8.34 ± 12.88
>75							
Male	9.19 ± 0.60	7.07 ± 0.64	6.83 ± 0.69	0.75 ± 0.04	0.74 ± 0.06	1.03 ± 0.13	−10.15 ± 5.09
Female	8.26 ± 0.53	6.18 ± 0.59	6.18 ± 0.42	0.76 ± 0.07	0.75 ± 0.05	1.02 ± 0.14	−9.61 ± 8.19

Abbreviations: MLW, medial‐lateral width of epicondyle; MPCH, medial posterior condyle height; LPCH, lateral posterior condyle height

### Univariate and Multivariate Analysis of Predicting Factors

Univariate analysis showed that age, sex, BMI, MLW, MPCH, LPCH, the ratio of MLW and MPCH, or the ratio of MLW and LPCH were not significantly associated with the successful retaining of PCL. A lower Insall–Salvati index (*P* = 0.001), preoperative diagnosis of rheumatoid arthritis (*P* = 0.008), and a higher severity of varus or valgus deformity (*P* = 0.027) were significantly associated with an increased risk of sacrificing the PCL (Table 1). In the multivariate analysis, patients with rheumatoid arthritis (*P* < 0.01), lower Insall–Salvati index (*P* < 0.01), and serious varus deformity (*P* = 0.012) were at an increased risk of sacrificing the PCL (Table 1).

### Outcomes of Sacrificing the PCL

Sacrificing PCL was not significantly associated with the size of the tibial and femoral components or the replacement of patella intraoperatively. However, we observed an increased risk of using thicker polyethylene insert among knees of the AS group compared with those in the CR group (*P* = 0.03) (Table 3).

**TABLE 3:** Comparison of implanted components between the retaining and recession of the posterior cruciate ligament (PCL)

	CR	AS	*P* value
The size of the femoral component			0.150
55	8	9	
57.5	66	20	
60	73	26	
62.5	31	16	
65	14	10	
67.5	19	7	
70/72.5	7	2	
The size of the tibial component			0.085
63	13	13	
67	76	26	
71	86	32	
75	28	9	
79/83	15	10	
The thickness of the polyethylene insert			0.003*
10	171	56	
12 and 14	47	34	
Patella replacement (yes/no)	122/95	47/43	0.548
Difference between MLW and the medial‐lateral lengths of the femoral components	19.37 ± 4.80	19.58 ± 5.04	0.720

* *p* < 0.05

Abbreviation: MLW, medial‐lateral width of epicondyle.

## Discussion

In this retrospective study, we found that patients with rheumatoid arthritis, a lower Insall–Salvati index, and a severe varus deformity were at a higher risk of sacrificing the PCL in a preoperatively planned CR TKA. The resection of PCL increased the risk of using a thicker polyethylene insert. To our knowledge, the current study was one of the few studies examining clinical and radiographic predictors of the successful preservation of the PCL.

### Preoperative Factors Predicting the Sacrificing of PCL

Although inflammatory arthritis, such as rheumatoid arthritis, was previously reported to be one of the contraindications for CR TKA,^15^ advances in implant design, surgical techniques, and rehabilitation have led to an expansion of the indications for CR TKA. Archibeck *et al*.^16^ and Dennis *et al*.^17^ suggested that CR TKA would yield satisfactory results in patients with rheumatoid arthritis. However, we found that patients with rheumatoid arthritis were more likely to convert from a CR‐type prosthesis to a AS‐type prosthesis intraoperatively. Patients with rheumatoid arthritis often have moderate to severe flexion contracture, severe contracture, or dysfunction of the PCL. It is challenging to achieve gap balancing in CR‐TKAs. Our results were consistent with that by Lombardi *et al*.^18^ who recommended CR‐TKA only for patients without severe coronal deformity and flexion contracture and PS‐TKA for patients with inflammatory arthritis.

Patients with a severe coronal deformity typically have some bone erosion and/or condylar dysplasia on the concave side of the deformity, while the convex side of the joint is affected by tension forces with stretched soft tissues, which may bring difficulty in gap balancing and necessitate resection of the PCL.^19^ A cadaveric study showed that there was significantly less change in flexion and extension gap after both medial and lateral releases with retention of the PCL.^20^ This finding supported that PS‐TKA may be more efficient for proper mediolateral (ML) balancing in knees with severe varus or valgus deformity. Laskin *et al*.^21^ reported that PS‐TKA provided superior results for patients with a varus deformity >15°. The authors observed significantly better postoperative alignment, flexion, and residual flexion contracture using a PS prosthesis than using a CR prosthesis. They also reported better results for a control group that used a PCL retaining prosthesis but did not exhibit preoperative severe deformity. Although Faris *et al*.^22^ and Merrill *et al*.^23^ reported that a CR prosthesis could be used as long as proper soft tissue balancing was performed at the time of surgery, they pointed out it was more difficult to correct knee angular deformities due to ligament imbalance and larger deformities intraoperatively. This is consistent with our findings that it was more difficult to achieve gap balancing in patients with varus deformities greater than 15°, which may result in the sacrifice of PCL.

Several studies have showed that patellar height is an important factor influencing intraoperative soft tissue balance at high flexion angles, especially in PS‐TKA.^24^ The primary function of PCL is to resist posterior tibial translation.^25^ It also acts to maintain a stable joint gap between the femur and tibia beyond 90° flexion.^2^ It has been reported that the PCL and the patellar tendon are almost parallel to the longitudinal axis of the tibia when the knee is flexed 90°.^26^Therefore, the patellar tendon is likely to be an important factor in maintaining the joint gap when the knee is flexed. Gejo *et al*.^27^ reported that patellar tendon strain increased gradually with knee flexion and that patellar tendon strain at 90° was associated with joint gap changes in PS‐TKA. Sasaki *et al*.^28^ found that patients with higher patellar positions had significantly larger component gaps than the lower group in flexion angle of 90° and 135° in PS‐TKA. They also found that the patella tendon strain might also be smaller in the higher patella group than in the lower patella group. These findings shed light upon a potential role of height of patella in CR TKA. In the current study we found that PCL was more likely to be preserved among patients with a higher patella position than those with a lower patella position. A hypothetic explanation for this finding is that a higher patellar position was related to a smaller patellar tendon strain, which made it easier to reach a suitable gap. However, we did not measure patellar tendon strain in this study. Further studies are warranted to investigate the underlying mechanism.

### Outcomes of Sacrificing the PCL

A change in the size of the tibial and femoral components may either affect or follow the resection of PCL. Bae *et al*.^7^ analyzed the factors affecting the conversion from CR‐ to PS‐TKA and found that the conversion rate from CR‐ to PS‐type prostheses was higher in patients with a small femoral component size. They attributed these results to morphologic characteristics of the distal femur and the aspect ratio (anteroposterior/ML ratio) in Asian populations.^29^ Asian patients usually have a small and narrow width of femoral condyles. When prostheses do not account for the changes in aspect ratio across the femoral condylar size, the femoral components may overhang smaller knees.^30^ This is not optimal because mediolateral overhang can result in irritation of the soft tissue or overstuffing of the joint space.^31^ In such cases additional distal femoral cutting may be required, which may further result in the resection of PCL because CR TKA allows less joint line elevation than PS TKA.^32^ In our study, no significant changes in MLW, MPCH, LPCH, the ratio of MLW and MPCH, or the ratio of MLW and LPCH were observed following the resection of PCL, suggesting that PCL may be preserved by careful femoral cutting. The relationship between the size of femoral components and the preservation of PCL still warrants further investigation.

We found that resection of PCL was associated with an increased risk of using a thicker polyethylene insert than the CR group. This is probably due to the increase in the medial‐lateral gap in both the flexion and extension gap after posterior cruciate ligament (PCL) resection.^33^

### Strengths and Limitations

Our study has strengths. First, our study, with few similar studies, added to the literature empirical evidence for preoperative predictors of sacrificing the PCL in planned CR TKA. Second, we identified risk factors from clinical information and radiographs, and provided data on the morphology of femoral condyle and patellar from a sample of Chinese patients. We acknowledge that this study has several limitations. First, it was a retrospective study of a consecutive series of TKAs using a single prosthesis. Most of the patients were female with osteoarthritic knees and varus deformities. This female predominance in the distribution of varus deformity limits the generalizability of our findings to other clinical scenarios. Second, the tibial and femoral component size, the final slope of the tibial cut surface, and the decision for conversion to the AS insert were determined by the planning and experience of a single surgeon, which may be susceptible to bias. Finally, some clinical and radiographic factors, such as the ROM, angle of flexion contracture, and posterior tibial slope angle, were not assessed due to a lack of information and radiographs. Further studies to address these limitations are needed.

## Conclusion

Patients with rheumatoid arthritis, a lower Insall–Salvati index, and a serious varus deformity were at a higher risk of sacrificing the PCL in a preoperatively planned CR‐TKA. The resection of PCL increased the risk of using a thicker polyethylene insert. These factors should be carefully taken into account for the appropriate selection of prosthesis type preoperatively.

## Author Contributions

Diange Zhou and Qiang Liu designed the study. Yi Wang and Liyi Zhang collected and analyzed the data and wrote the first draft of manuscript. Jianhao Lin and Dan Xing participated in the data collection, analysis, and interpretation. All authors read and approved the final manuscript.

## Data Availability

Data will be available upon request by the corresponding authors.
